# LncRNA in tumorigenesis of non-small-cell lung cancer: From bench to bedside

**DOI:** 10.1038/s41420-022-01157-4

**Published:** 2022-08-13

**Authors:** Qin Hu, Huiyun Ma, Hongyu Chen, Zhouwei Zhang, Qun Xue

**Affiliations:** 1 Research Center of Clinical Medicine, Affiliated Hospital of Nantong University, Nantong, China; 2 Medical School of Nantong University, Nantong, China; 3 Department of Cardiothoracic Surgery, Affiliated Hospital of Nantong University, Medical School of Nantong University, Nantong, China

**Keywords:** Non-small-cell lung cancer, Non-small-cell lung cancer

## Abstract

Lung cancer has been one of the leading causes of cancer-related death worldwide, and non-small-cell lung cancer (NSCLC) accounts for the majority of lung cancer morbidity, yet the pathogenesis of NSCLC has not been fully elucidated. Recently, long-chain non-coding RNA (lncRNA) has attracted widespread attention. LncRNA is a type of non-coding RNA whose transcript length exceeds 200 nucleotides. After constant research, academics updated their understanding of lncRNA, especially its role in the biological processes of cancer cells, including epigenetic regulation, cell proliferation, and cell differentiation. Notably, examination of lncRNAs could serve as potential hallmarks for clinicopathological features, long-term prognosis, and drug sensitivity. Therefore, it is necessary to explore the functions of lncRNA in NSCLC and innovate potential strategies against NSCLC based on lncRNA-related research. Herein, we reviewed the functions of lncRNA in the occurrence, diagnosis, treatment, and prognosis of NSCLC, which not only help promote a comprehensive view of lncRNA in NSCLC, but also shed light on the potential of lncRNA-based diagnosis and treatment of NSCLC.

## Facts

- LncRNA plays a biological role in NSCLC through different pathways, such as lncRNA-protein interaction, lncRNA-ceRNA network, lncRNA-miRNA-mRNA silencing, and binding to DNA in cis and binding to promoter regions of encoding genes.
- LncRNAs regulate the proliferation, invasion, and migration of cancer cells in NSCLC through their interactions with miRNAs, WNT pathways, exosomal lncRNAs and LCSCs, thus leading to tumor development and change.
- Detection of lncRNA in tissues and blood is beneficial to the diagnosis and treatment of NSCLC.

## Open questions

- How to target lncRNA-mediated oncogenic mechanisms?
- To find a method to construct highly specific tumor-associated lncRNA.
- Highly sophisticated techniques are expected to screen for lncRNA populations that are widely used in tumor diagnosis and treatment.

## Introduction

Lung cancer is a malignant tumor with extremely high morbidity and mortality in the world. According to the International Agency for Research on Cancer (IARC) of the World Health Organization [1], the number of new lung cancer cases worldwide reached to 1.82 million in 2018, ranking the first in the incidence of all malignant tumors. Although the worldwide morbidity has decreased, the situation is still serious in China [2, 3]. Non-small-cell lung cancer (NSCLC) is the main type of lung cancer, accounting for about 80% of all cases. Based on histological characteristics, NSCLC is mainly divided into squamous cell carcinoma, adenocarcinoma, adenosquamous carcinoma, large cell lung cancer, and sarcomatoid carcinoma [4]. Lack of typical symptoms leads to the sticky circumstance that some patients are detected with metastatic NSCLC at first diagnosis, so the prognosis is poor and the 5-year survival rate is usually less than 20%, which is a serious threat to human life and health [5]. Therefore, studying the mechanism of NSCLC tumorigenesis and progression is crucial for the diagnosis and treatment of NSCLC. With the development of molecular biology technologies, long-chain non-coding RNA (long non-coding RNA, lncRNA) has gradually become a research hotspot. LncRNAs take part in the initiation, and progression of cancer by various mechanisms. Moreover, different lncRNAs are also engaged in regulating sensitivities to chemotherapy, targeted therapy, and even radiotherapy [6, 7]. In this review, we summarized the role of lncRNAs in lung cancer progression, discussed the underlying mechanisms driving the related biological process, and prospected the potential application targeting lncRNAs in NSCLC.

## Overview of lncRNA

LncRNA is a type of non-coding RNA, named for its transcript of over 200 nucleotides and lack of protein-coding ability. LncRNA regulates a variety of important biological processes, including epigenetic regulation, cell division, and cell differentiation [8, 9]. The research found that lncRNAs are dysregulated and aberrantly expressed in a variety of tumors [10]. LncRNA has binary roles in NSCLC by binding to promoter regions of certain genes, mediating chromatin remodeling, regulating histone modification, and interfering with the biological function of transcription factors. Moreover, lncRNA often binds to specific proteins or works as the precursor molecules of small molecule RNA [11, 12]. Therefore, it has a variety of complex regulatory networks. Emerging investigation on lncRNA in NSCLC shows that lncRNA can affect a variety of signaling pathways and play a pivotal role in the initiation and progression of NSCLC.

Increasing evidence showed that LncRNA played biological roles through various and distinct pathways in different systems. LncRNA mainly functions in the following ways: (1) by lncRNA-protein interaction; (2) by lncRNA-ceRNA network; (3) by lncRNA-miRNA-mRNA silencing; (4) by binding to DNA in cis; (5) by binding to promoter regions of encoding genes. Therefore, it is widely thought that lncRNA can affect a variety of signaling pathways in NSCLC [13, 14].

## The association between abnormal lncRNA expressions and NSCLC development

**Fig. 1:**
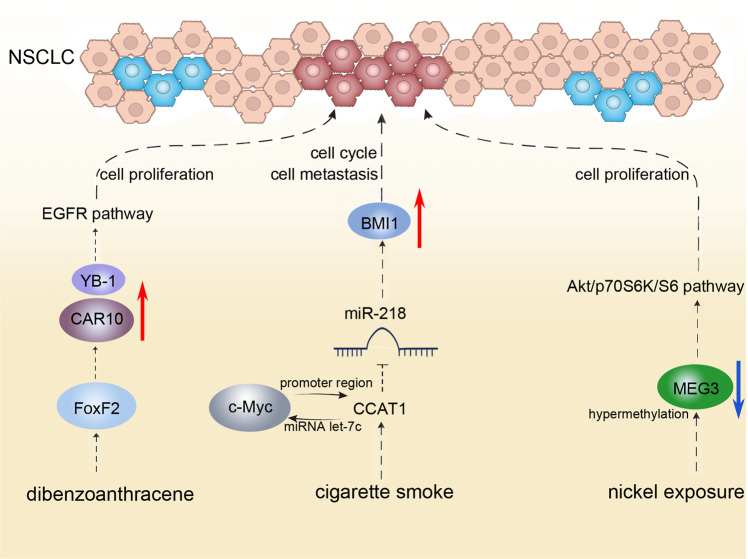
Schematic of LncRNA-based regimen in NSCLC.

It was reported that 93.75% of lung cancer in China was associated with smoking, the leading cause of squamous cell carcinoma [15]. Abnormal lncRNA expressions were associated with the development of NSCLC. LncRNA CCAT1 was reported to be involved in this pathological process after exposure to cigarette smoke extracts, CCAT1 inhibits miR-218 transcription and promotes BMI1 expression, leading to the pro-tumoral phenotype of NSCLC, activating cell cycle and facilitating cancer metastasis [16]. Further research pointed out a positive feedback loop of c-Myc and CCAT1, which also explains the lncRNA CCAT1-mediated NSCLC tumorigenesis [17]. The oncogenic transcription factor c-Myc can activate the expression of CCAT1 by binding to the promoter region of CCAT1, and CCAT1 can in turn promote the expression of c-Myc by binding free miRNA let-7c [17]. Another environmental factor of NSCLC occurrence is air pollution. Studies have shown that 12.8% of lung cancer mortality worldwide is attributable to air pollution caused by human production [18]. A regional study identified in NSCLC patients abnormal levels of lncRNAs, among which lncRNA CAR intergenic 10 (CAR10) possessed a significantly high expression compared with patients from other regions [18]. Mechanistically, for patients in high coal-consumption regions, the level of dibenzoanthracene, a member of the polycyclic aromatic hydrocarbon family, increase, which therefore promoted the transcription of lncRNA CAR10 by FoxF2 in lung cancer epithelial cells, and the expression of CAR10 upregulated EGFR signaling pathway thereby promoting cell proliferation and inducing tumorigenesis [19, 20]. Moreover, lncRNAs also take part in the mechanism that PM 2.5 exposure increases the risk of lung cancer. High dose exposure increases reactive oxygen species (ROS) level and upregulates the expression of lncRNA loc146880 in NSCLC cells; besides, it also contributes to cancer cell autophagy, which further enhances the migration and invasion ability of tumor cells. Occupational exposure is also an important risk factor for the occurrence of NSCLC. For example, nickel exposure downregulated MEG3 expression which is an anti-proliferative lncRNA in cancer cells [21–25]. The underlying mechanism may be that nickel exposure leads to hypermethylation and expression inhibition of the MEG3 promoter region, leading to activation of the Akt/p70S6K/S6 pathway, thereby causing cancerization of human bronchial epithelial cells. The above investigation indicated that lncRNA plays an important role in the initiation of NSCLC induced by exposure to environmental risk factors. Research on these factors and pathways will help to study the biological mechanism of NSCLC and provide clues for the prevention and treatment of NSCLC (see Fig. 1).

## Clinical application of lncRNA

### lncRNA related to the diagnosis of NSCLC

Although lung cancer diagnosis technology continues to improve, some scholars have pointed out that 40% of NSCLC patients are still diagnosed with locally advanced or advanced lung cancer that usually cannot be surgically removed, so there is an urgent need to explore an effective, convenient and economical diagnostic method for the early diagnosis, early detection and early treatment of lung cancer including NSCLC [18, 26]. In recent years, research on lncRNA has found that the abnormal expression of lncRNA in NSCLC cells is closely related to the occurrence and progression of NSCLC. With the three important features—stability, specificity, and easy availability—lncRNA may become a potential bio-diagnostic marker for NSCLC. Studying the expression profiles of these lncRNAs in NSCLC tissues and blood will help to develop accurate diagnostic markers and improve the detection rate of NSCLC patients.

#### Research on lncRNA in tumor tissues

Many studies have found that the abnormal expression of lncRNA in lung cancer tissues is closely related to the occurrence and development of tumors. Su et al. studied the effect of the lncRNA PRAL (P53 regulatory correlation lncRNA) in lung cancer [27]. The study found significantly decreased expression of P53 and the P53-related lncRNA PRAL in lung cancer lesions compared with that in adjacent normal tissues, whereas ectopic expression of PRAL in NSCLC cell lines remarkably inhibited the proliferation of tumor cells. This indicates that low expression of lncRNA PRAL in primary lesions may be used as a marker for the diagnosis of NSCLC.

Besides, researchers reported that lncRNA linc00312 located at 3p25.3 was downregulated in NSCLC tumor samples and correlated with the clinicopathological status of NSCLC patients. [28, 29]. Further experiments found a positive correlation between the expression of linc00312 and the transcription factor HOXA5 which was a tumor-suppressor involved in several cancers progression [29]. While HOXA5 is a transcriptional factor promoting cancer cell proliferation, this research indicated that linc00312 may play an important role in cell proliferation and tissue invasion. Unlike other tumors, the cancer-promoting function of HOXA5 in lung cancer needs further study.

Recently, urothelial carcinoma-associated gene 1 (UCA1) has been confirmed as an oncogene, and it is believed that the dysregulated expression of UCA1 is closely related to the initiation and development of tumors [30, 31]. Studies have found that UCA1 is highly expressed in NSCLC tissues, and silencing UCA1 will reduce the proliferation ability of lung cancer cells including NSCLC [30]. Therefore, the above research suggested the predictive value of ectopic lncRNA expression in NSCLC tissues, and indicated that lncRNA could serve as bio-diagnostic markers and provide new indicators for the diagnosis of NSCLC.

#### Free lncRNA in liquid biopsy

Recently, circulating long non-coding RNA has become the focus of tumor biomarker research, and studies suggested that it has a very important significance for the diagnosis of cancer. Tang et al. used lncRNA chips to analyze and screen out potential NSCLC biomarkers in the circulation and found that the expression of three lncRNAs (RP11-397D12.4, AC007403.1, ERICH1-AS1) were all up-regulated, and established a predictive model based on these three abnormally expressing lncRNA [32]. The above work suggested that these three lncRNAs were expected to be potential biomarkers for the early diagnosis of NSCLC.

Similarly, Hu et al. found differentiated expression of another three circulating lncRNAs: SPRY4-IT1, ANRIL, and NEAT1 in tumor tissues and identified the relationship with clinical prognosis based on ROC analysis [14]. Combined, the performance of lncRNA as predictive biomarkers for clinical prognosis will be greatly improved, the sensitivity was 82.8%, and the specificity reached 92.3%, and the area under the curve (AUC) was 0.876. The above research proposed the detection of circulating lncRNA as a strategy in liquid biopsy. Additionally, the heterogeneity of lncRNA was also reported and certain single lncRNA abnormalities also contribute to the diagnosis of NSCLC [33].

Studies have found that the expression of lncRNA growth arrest-specific transcript 5 (GAS5) is low in lung cancer tissues compared with adjacent normal tissues. Liang et al. found that in the plasma of NSCLC patients, GAS5 is in a stable state with a low expression [33]. Its diagnostic sensitivity and specificity are 82.2 and 72.7%, respectively, and the AUC is 0.832, which confirms the relationship between GAS5 and NSCLC diagnosis. UCA1 expression in the plasma of NSCLC patients was found significantly increased, which is consistent with the expression in tumor tissues, with an AUC of 0.886 [31]. Therefore, plasma UCA1 can also be used as a potential biomarker for the diagnosis of NSCLC, which can improve the screening efficiency of NSCLC.

### LncRNA related to NSCLC treatment

LncRNA plays an important role in the occurrence and development of NSCLC, and the corresponding molecular targeted therapy is also under continuous development. Similar to other targeted therapies, lncRNA-based target therapy includes silencing, blocking, destroying carcinogenic lncRNA, or transferring the tumor suppressor lncRNA into specifically targeted cells. LncRNA has versatility, providing it with a promising application potential in the treatment of diseases, with the deepening of the understanding of lncRNA in the development of lung cancer, its application in the treatment of NSCLC will become more and more extensive. Only when we fully and thoroughly understand the biological mechanism of lncRNA functions in NSCLC can we effectively accelerate the production of new therapies. Next, we review the diverse mechanism of lncRNA functions in NSCLC.

#### lncRNAs interact with microRNAs to promote the proliferation, invasion, and metastasis of cancer cells in NSCLC

**Fig. 2:**
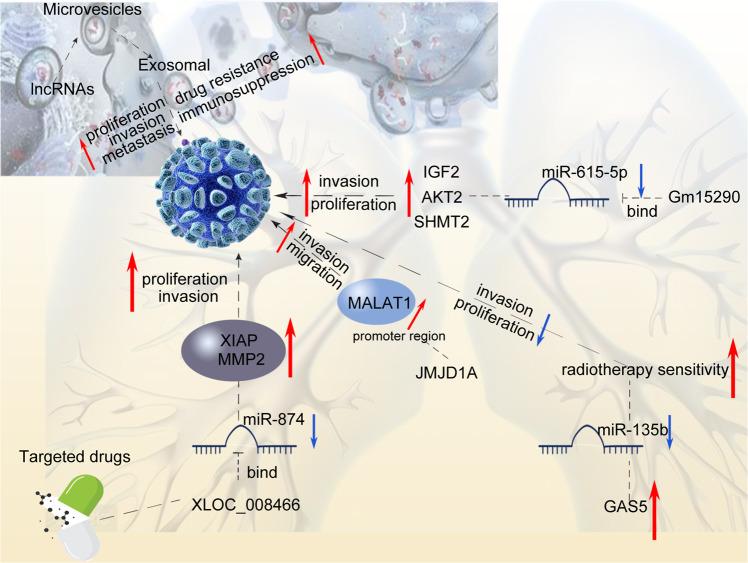
Schematic of LncRNAs interact with microRNAs regimen in NSCLC.

In NSCLC, studies have shown that many oncogenic lncRNAs function in tumors through interaction with microRNAs [11]. Yang et al. found that lncRNA XLOC_008466 is highly expressed in NSCLC patients. Inhibiting XLOC_008466 expression decreased the proliferation and invasion, but promoted the apoptosis of NSCLC [34]. Studies have pointed out that XLOC_008466 functions similarly to ceRNA and can directly bind and downregulate miR-874, which will increase the expression of the miR-874 downstream targets, MMP2 and XIAP. Targeted drugs against XLOC_008466can reduce the proliferation and invasion of NSCLC cells Another study reported a lncRNA Gm15290/miR-615-5p/targeted genes axis in NSCLC and suggested Gm15290 inhibition as a potential treatment of NSCLC [35]. Small molecule inhibitors are possible choices for lncRNA inhibition. For instance, JMJD1A induces cell migration and invasion by upregulating the expression of the long noncoding RNA MALAT1. The small molecule JMJD1A inhibitor DMOG suppresses neuroblastoma cell migration and invasion [36]. Xue et al. found that GAS5 expression was downregulated in NSCLC tissues and cells, and the expression of miR-135b was upregulated [37]. The experimental results showed that high expression of GAS5 and low expression of miR-135b can significantly reduce the survival rate of NSCLC cells under irradiation and improve radiotherapy sensitivity, at the same time, which can significantly inhibit the occurrence of tumors by inhibiting the proliferation and invasion of tumor cells (see Fig. 2).

#### LncRNAs involve in the regulation of signaling pathways in NSCLC

The occurrence and progression of tumors are inseparable from the abnormal activation of multiple signaling pathways in tumor cells. Studying how lncRNAs regulate the malignant behaviors of tumor cells is an important process to understand the underlying mechanism of tumor initiation and is very important for the further development of new therapeutic targets. LncRNA CBR3-AS1 contributes to the proliferation, migration, and invasion of lung adenocarcinoma cells via activating the Wnt/β-catenin signaling pathway [20]. And LncRNA FEZF1-AS1 promotes epithelial-mesenchymal transition (EMT) of NSCLC cells by regulating the WNT pathway [38]. Besides, lncRNA insulin growth factor 2 antisense(IGF2AS) is not only an imprinted gene in Wilms tumors involved in the transcription and translation of a variety of proteins but also a low-expressing lncRNA in NSCLC tissues, which is closely related to the overall survival of patients and a potent inhibitor to the migration of NSCLC cells [39]. Unlike mouse IGF-2AS, human IGF-2AS likely encodes a putative peptide consisting of 273 amino acids [40, 41]. IGF-2AS transcripts are mainly present in the cytoplasm and are associated with polysomes, suggesting that the mechanism by which IGF-2AS is involved in tumor progression may be related to the regulation of protein translation [40, 41].

LncRNAs took part in tumorigenesis by regulating the mutation frequency and inactivating tumor suppressor genes such as LKB1 in NSCLC, while LKB1 inactivation leads to linc00473 expression in turn. High expression of LINC00473 is associated with poor prognosis, and the survival of LKB1-knockout NSCLC cells is dependent on the expression of LINC00473. Mechanistically, LKB1 inactivation and subsequent cyclic AMP response element binding protein (CREB)/CREB-regulated transcriptional coactivator (CRTC) activation induces the expression of LINC00473. LINC00473 is a nuclear lncRNA and interacts with NONO to promote CRTC/CREB-mediated transcription [42]. This regulating network provides a new direction for the targeted therapy of NSCLC. HOTAIR is a kind of lncRNA with trans- transcriptional regulation, which can negatively regulate chromosome transcription, reorganize chromatin and promote tumor progression. A study on glioblastoma found that HOTAIR expression was associated with tumor cell proliferation and migration [43]. Mechanistically, murine models revealed that lncRNA HOTAIR was downregulated after radiotherapy, resulting in changes in β-catenin signal transduction [43]. In addition, cell line experiments demonstrated that lncRNA MEG3 could serve as a predictor of palbociclib sensitivity in NSCLC in that the expression of MEG3 was positively correlated with phosphorylated Rb protein and suppressed cell cycle [44]. It can be seen that inhibitory lncRNA also has a wide range of application prospects in the treatment of NSCLC, but further research and exploration are needed for better clinical application.

**Fig. 3:**
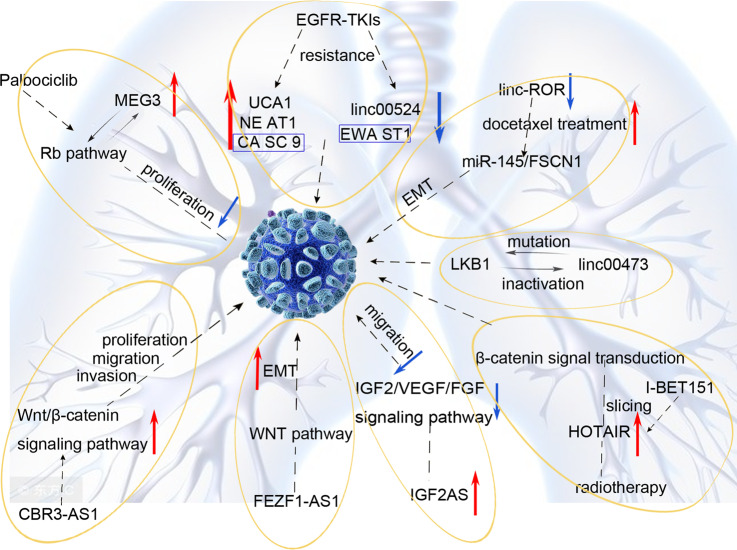
Schematic of LncRNAs regulate signaling pathways regimen in NSCLC.

Studies have found that EGFR mutations occur in over 20% of NSCLC patients, which suggests sensitivity to epidermal growth factor receptor tyrosine kinase inhibitors (EGFR-TKIs) such as erlotinib and gefitinib [45, 46], but about 10% of patients will develop resistance within 10–16 months [47]. Recently, some scholars pointed out that lncRNA is involved in the resistance of EGFR-TKIs with a variety of lncRNAs abnormally expressed in EGFR-TKIs resistant cells [48]. Based on cell line experiments, five abnormally expressed lncRNAs were found in the gefitinib-resistant NSCLC cell line, including three highly expressed lncRNAs (UCA1, NE AT1, CA SC 9) and two low-expressed lncRNAs (EWA ST1, linc00524). Further study on the mechanism of lncRNA in EGFR-TKIs resistance found that co-expression of CASC9 and EWAST1 was involved in several pathways, including the regulation of cell growth, apoptosis, chromatin assembly, and other pathways, which in turn affected drug sensitivity. Lung adenocarcinoma is the main type of NSCLC and has high resistance to chemotherapeutics. Pan et al. studied the surgical specimens of patients with lung adenocarcinoma who are resistant to docetaxel and found elevated expression of linc-ROR in docetaxel-resistant patients [49]. In vivo experiments have shown that downregulation of linc-ROR expression will increase docetaxel sensitivity by regulating the EMT process through linc-ROR/miR-145/FSCN1 axis (see Fig. 3).

#### Exosome-derived lncRNAs in NSCLC

At present, a large number of research have proved that under pathological conditions, especially tumor diseases, the expression levels of many exosomal lncRNAs are significantly different from those in normal control samples. Many types of research suggested that exosomal lncRNA differs between normal and malignant tissues, which indicates that exosomes can selectively package, secrete and transfer specific lncRNAs and play the corresponding biological function [50]. Exosomal lncRNA research is an important part of tumor biology. It can participate in tumor cell proliferation, invasion, metastasis, angiogenesis, drug resistance, and immunosuppressive microenvironment [50]. Exosomal lncRNA can be derived from tumor cells, tumor-associated macrophages, and fibroblasts, and acts on specific target cells through signal transmission between malignant cells and non-malignant cells, remodeling the tumor microenvironment, and promoting tumor cell proliferation, invasion and metastasis. In addition, exosomal lncRNA has been proven to regulate antigen presentation, affect the cytotoxicity of immune cells, and induce the apoptosis of related effector cells [13]. Due to the above characteristics of exosomal lncRNA, it is currently generally believed that it can be used as an early diagnosis and monitoring effect of tumors. And effective intervention on them may help to find new targets for the treatment of lung cancer.

Studies have shown that exosomal lncRNA has important clinical significance for the early diagnosis of lung cancer. A high expression level of UFC1 in serum and serum exosomes was detected to be associated with tumor invasion in NSCLC patients [51]. Other researchers quantitatively analyzed the serum exosomal lncRNA of NSCLC patients and healthy controls, and indicated thatTBILA and AGAP2-AS1 have diagnostic efficiency for early NSCLC patients [52]. And the combination of these two exosomal lncRNAs and the widely used clinical serum tumor biomarker (CA21-1) can further improve the diagnosis accuracy. An analysis on 64 NSCLC patients and 40 healthy subjects showed that compared with healthy controls, the expression level of GAS5 in NSCLC patients was downregulated [53]. In addition, its low expression was related to tumor volume and clinicopathological stage. Therefore, GAS5 can be used as a marker for the early diagnosis of NSCLC. Zhang et al. found that exosomal MALAT-1 is highly expressed in NSCLC patients, especially the expression level of exosomal MALAT-1 and tumor staging [54]. It is positively correlated with lymphatic metastasis and can be used as a liquid biopsy marker for the diagnosis and prognosis of NSCLC.

#### LncRNA and lung cancer stem cells (LCSCs)

Refractory disease with constant drug resistance may be closely related to the existence of LCSCs, a population with self-renewal and differentiation abilities. More and more studies have shown that lncRNA exerts an effect on LCSC. Sun et al. found that LncRNA CCAT1 contributed to LCSC-induced tumor progression directly and indirectly. CCAT1 not only induces symmetrical division of LCSCs, but also interacts with the Wnt signaling pathway which is conducive to the expansion of LCSCs, leading to recurrence and treatment failure, and this CCAT1-associated mechanism could be reversed by Let-7c [55]. Both the restoration of Let-7 and the treatment of Wnt signaling inhibitor Axitinib can effectively reverse the role of CCAT1 in promoting symmetrical division and self-renewal. Therefore, LncRNA CCAT1 regulates the division of LCSCs through the CCAT1/Let-7c/Wnt regulatory axis. This report demonstrated the oncogenic role of CCAT1 and indicated a new mechanism behind the Wnt signaling pathway. Stimulating the asymmetric splitting of LCSCs by delivering Let-7c or the inhibitor Axitinib may represent a prospective strategy for the treatment of lung cancer patients.

**Fig. 4:**
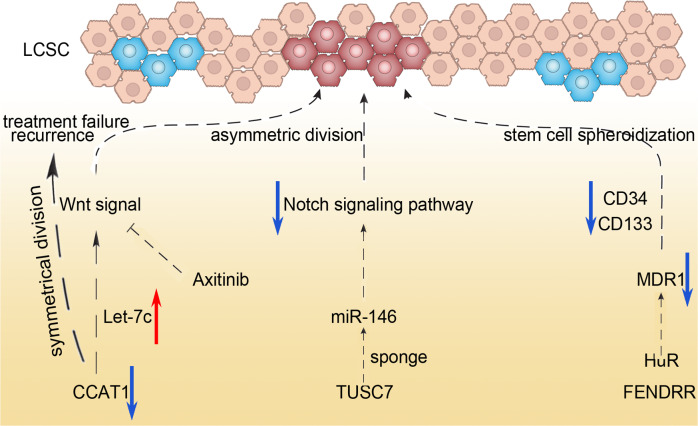
Schematic of LncRNA and Lung cancer stem cells regimen in NSCLC.

At the same time, the researchers also found that LncRNA TUSC7 inhibits the activation of the Notch signaling pathway through sponge adsorption of MiR-146, thereby inducing asymmetric division of LCSCs and inhibiting the renewal ability of LCSCs [56]. Therefore, it can be seen that LncRNA can affect its self-turnover ability by regulating the way of division of LCSCs. Xu et al. found that both lncRNA FENDRR and HuR can bind to the 3′untranslated region (3′UTR) of multidrug resistance gene 1 (MDR1), and they exhibit opposite effects and compete with each other. HuR belongs to the family of RNA-binding proteins (RBPs) [57]. As an epigenetic regulator, it can positively regulate the stability of RNA involved in tumor progression. MDR1 is a key mediator of CSC chemotherapy resistance [58, 59]. Therefore, FENDRR directly and specifically binds to MDR1 3′UTR, hindering the interaction of HuR and MDR1 3′UTR, thereby reducing the expression of MDR1 to weaken the expressions of the LCSCs stemness markers including CD34 and CD133 and reducing the ability of stem cell spheroidization (see Fig. 4).

### LncRNAs related to the prognosis of NSCLC

Due to the atypical clinical manifestations in the early stage of NSCLC patients, some patients are detected with metastasis at first diagnosis, predicting a poor prognosis in the long term. As mentioned above, a variety of lncRNAs are related to the diagnosis and treatment of NSCLC, which helps improve the prognosis of patients. Meanwhile, the related lncRNAs are also expected to become an important factor in evaluating prognosis [60]. Studies have found that the expression of SPRY4-IT1 is an independent risk factor for the prognosis of NSCLC (*P* = 0.009) and a significant predictor of clinicopathological features [61]. The latest study found that lncRNA NEAT1 and MALAT1 are highly expressed in NSCLC tissues. It can also promote the occurrence and development of tumors through the regulation of Oct4, and the high expressions of Oct4, NEAT1, and MALAT1 are related to the poor prognosis of NSCLC patients, and its HR = 2.78 (95%CI: 1.21–6.42) [9]. To explore the relationship between the expression of lncRNA and prognosis in lung adenocarcinoma, Tang et al. distinguished five abnormally expressed lncRNAs, including ZNF503-AS1, CYP4F26P, RP11-108M12.3, RP11-38M8.1, and RP11- 54H7.4 [62]. Comprehensive analysis based on these 5 lncRNAs predicts the 5-year survival of NSCLC patients In addition, many other studies indicated the potential application of lncRNA to evaluate therapy efficacy and clinical prognosis [63]. Therefore, the researches on these lncRNAs are beneficial in assessing the prognosis of NSCLC patients and provides new research directions for improving the prognosis (see Table 1).

**Table 1:** Functions of different lncRNAs in lung cancer.

lncRNA	Functions in lung cancer	References
lncRNA PRAL	Decreased expression of P53.	[28]
	Inhibited the proliferation of tumor cells.	[28]
lncRNA linc00312	Correlated with the clinicopathological status of NSCLC patients	[29]
	Regulate the expression of transcription factor HOXA5	[30]
Urothelial carcinoma-associated gene 1 (UCA1)	Related to the initiation and development of tumors	[31, 32]
RP11-397D12.4, AC007403.1, ERICH1-AS1	A predictive model based on these three abnormally expressing lncRNA	[33]
SPRY4-IT1, ANRIL, and NEAT1	The relationship with clinical prognosis	[7, 12, 61]
Growth arrest-specific transcript 5 (GAS5)	A potential biomarker for the diagnosis of NSCLC	[34]
MALAT1	The regulation of Oct4	[7]
ZNF503-AS1, CYP4F26P, RP11-108M12.3, RP11-38M8.1 and RP11- 54H7.4	Predicts the 5-year survival of NSCLC	[62]

## Conclusion and future perspectives

In recent years, the incidence and death of lung cancer have been increasing, and research on the mechanism of lung cancer is also ongoing. With the rapid development and application of multi-omics sequencing technology, the research on related lncRNAs in the development of lung cancer is also deepening. LncRNAs play an important role in NSCLC induced by exposure to environmentally hazardous substances. According to reports, lncRNAs including CCAT1, CAR10, and MEG3 can induce malignant transformation of bronchial epithelial cells and initiate tumors. Previous studies have proved that a variety of lncRNAs are abnormally expressed in NSCLC tissues and the circulating system of NSCLC patients. These lncRNAs can be used as markers for the diagnosis of NSCLC. Unlike miRNA, the expression level of lncRNA can better reflect the disease status; at the same time, the expression pattern of lncRNA is highly specific, suggesting that its expression status can be used for disease diagnosis or classification. Among them, UCA1, as an important factor in the occurrence and development of tumors, is abnormally expressed in tumor tissues and blood, making it an effective diagnostic marker for NSCLC. Still, the stability and effectiveness of its expression in tissues and blood need to be further studied. In addition, different types of lncRNAs can also be used as prognostic markers of NSCLC. By studying the abnormal expression of lncRNAs, we not only polish the understanding of the malignant process during tumor development but also increase the possibility for better clinical treatments of NSCLC patients. It can be seen that the study of these lncRNAs provides a new research direction for the diagnosis and prognosis of NSCLC. With the continuous development of treatment methods, targeted therapy has become an effective treatment method for cancer, and lncRNA plays an important role in future development in this area. More and more lncRNAs have been discovered, including carcinogenic lncRNAs, such as XLOC_008466, Gm15290, linc00473, etc. Their overexpression can promote the proliferation, migration, invasion, and metastasis of NSCLCcells. Besides that, there are also anti-tumoral lncRNAs such as IGF2AS, MEG3, and oncogenic lncRNA HOTAIR. In addition, the genetic polymorphism of lncRNA is related to chemotherapy sensitivity, and can also be used as a biomarker for pretreatment assessment of lung cancer patients to improve chemotherapy efficacy [64, 65]. These lncRNAs are not only expected to become effective targets for therapeutic drugs, but also play an important role in predicting the sensitivity of radiotherapy and chemotherapy. Researching these lncRNAs will help to understand their mechanisms in the development of NSCLC, leading to effective treatment for patients with NSCLC. With the continuous deepening of the research and development of anti-tumor drugs, the research on small molecule compounds has made some progress, which brings broad prospects for the development of targeted therapy. However, there is still a long way to go before we clarify the specific application of lncRNA in NSCLC because of the limited research on lncRNA targeted therapy. In the future, with the advancement of new technologies, such as spatial single-cell transcriptome sequencing, the expression changes of LncRNAs in lung cancer will be further clearly observed. In addition to the altered expression of lncRNA in tumor cells, it may also be expressed in other cells in the tumor microenvironment, such as tumor fibroblasts. LncRNA may also play a role in the interaction between tumor cells and other cells, which needs to be further studied. It is necessary to explore the relationship and interaction between lncRNA and the traditional treatment of lung cancer, which could shed light on novel methods for treating NSCLC.
